# 

**Published:** 1892-03

**Authors:** 


### THE BRISTOL

### Medico-Chirurgical Journal.

A JOURNAL OF THE MEDICAL SCIENCES FOR THE WEST OF ENGLAND AND SOUTH WALES

*PUBLISHED UNDER THE AUSPICES OF*

*THE BRISTOL MEDICO-CHIRURGICAL SOCIETY.*

EDITOR:

R. SHINGLETON SMITH, M.D., B.Sc.

WITH WHOM ARE ASSOCIATED

BARCLAY J. BARON, M.B., C.M.

J. MICHELL CLARKE, M.A., M.D.

F. R. CROSS, M.B.

W. H. HARSANT.

ASSISTANT-EDITOR:

L. M. GRIFFITHS.

SURGEON GENL'S OFFICE
147430
LIBRARY.

"Scire est nescire, nisi id me
Scire alius sciret."

*VOL. X.*

BRISTOL: J. W. ARROWSMITH;
LONDON: J. & A. CHURCHILL, 11 NEW BURLINGTON STREET.
1892.

WI
BR323

J.W. Arrowsmith

Publisher Bristol

33750

### CONTENTS OF VOLUME X.

Page

A Recent Outbreak of Rötheln. By WILLIAM JOHNSTONE FYFFE, M.D. Dub.... ... ... ... ... ... ... ... ... ... ... 1

On a Rare Form of Spina Bifida (Syringo-Myelocele). By CHARLES A. MORTON, F.R.C.S. Eng. ... ... ... ... ... ... 14

The Quantitative Estimation of the Knee-Jerk. By EWEN J. MACLEAN, M.D., C.M. Edin. ... ... ... ... ... ... ... 20

Extra-Uterine Pregnancy. By A. E. AUST LAWRENCE, M.D., C.M. Aberd. ... ... ... ... ... ... ... ... ... ... ... 73

Hay Fever and Hay Asthma. By P. WATSON WILLIAMS, M.D. Lond. ... ... ... ... ... ... ... ... ... ... ... ... ... 84

Forceps Delivery during the last Fifty Years. By JOSEPH GRIFFITHS SWAYNE, M.D. Lond. ... ... ... ... ... ... ... 153

A Case of Large Ovarian Tumour in a Young Girl. By J. MACPHERSON LAWRIE, M.D., C.M. Glasg. ... ... ... ... ... 186

A Case of Multiple Symmetrical Joint Lesions. By CHARLES A. MORTON, F.R.C.S. Eng. ... ... ... ... ... ... ... ... ... 189

The Teachings of Failure. By E. MARKHAM SKERRITT, B.A., M.D. Lond., F.R.C.P. ... ... ... ... ... ... ... ... ... 229

Two Cases of Locomotor Ataxy with Charcot's Joint Disease. By HENRY DAVY, M.D. Lond., and ARTHUR G. BLOMFIELD, M.D. Aberd. ... ... ... ... ... ... ... ... ... ... ... 249

Health-Resorts in the West of England and South Wales.
Tenby. By John Griffith Lock, M.A. Cantab., M.R.C.S. Eng., L.R.C.P. Ed., L.S.A. ... ... ... ... ... ... ... 97
Bath. By A. B. Brabazon, M.D. Aberd. ... ... ... ... 254

The Early History of the Bristol Medical School. By Augustin Prichard, F.R.C.S. Eng. ... ... ... ... ... ... ... ... 264

Progress of the Medical Sciences ... ... 26, 107, 195, 292

Reviews of Books ... ... ... ... ... ... 45, 127, 211, 311

Notes on Preparations for the Sick ... ... 57, 142, 222, 327

Meetings of Societies ... ... ... ... ... ... 57, 144, 329

The Library of the Bristol Medico-Chirurgical Society ... ... ... ... ... ... ... ... 62, 146, 223, 332

Obituary ... ... ... ... ... ... ... ... ... ... ... ... ... 67

International Congress of Gynæcology and Obstetrics ... 68

Alvarenga Prize of the College of Physicians of Philadelphia ... ... ... ... ... ... ... ... ... ... ... ... 69

Presentation to Sir George Buchanan, F.R.S. ... ... ... 149

The Jefferson Medical College of Philadelphia ... ... 150

Scraps ... ... ... ... ... ... ... ... ... 69, 150, 227, 339

### Bristol Medico-Chirurgical Society.

---

### PAST PRESIDENTS.

1874—75. FREDERICK BRITTAN, M.D., B.A. Dub.

1875—76. ROBERT WILLIAM COE.

1876—77. SAMUEL MARTYN, M.D. Ed.

1877—78. AUGUSTIN PRICHARD, M.D. Berlin.

1878—79. HENRY EDWARD FRIPP, M.D. Würzburg.

1879—80. WILLIAM MICHELL CLARKE.

1880—81. JOSEPH GRIFFITHS SWAYNE, M.D. Lond.

1881—82. EDWARD LONG FOX, M.D. Oxon.

1882—83. JAMES GEORGE DAVEY, M.D. St. And.

1883—84. WILLIAM JOHNSTONE FYFFE, M.D. Dub.

1884—85. GEORGE FORSTER BURDER, M.D. Aberd.

1885—86. WILLIAM HENRY SPENCER, M.D., M.A. Cantab.

1886—87. FRANCIS POOLE LANSDOWN.

1887—88. LEMUEL MATTHEWS GRIFFITHS.

1888—89. ROBERT SHINGLETON SMITH, M.D., B.Sc. Lond.

1889—90. NELSON CONGREVE DOBSON.

1890—91. SAMUEL HENRY SWAYNE.

1892.

### LIST OF SUBSCRIBERS

TO THE

### Bristol Medico-Chirurgical Journal.

*Those marked * are Members of the Society.*

| | |
|---|---|
| Abercrombie, John, M.D. Cantab. ... ... ... | 23 Upper Wimpole Street, London, W. |
| Ackland, J. McKno ... ... ... ... ... ... | 24 Southernhay, Exeter. |
| *Ackland, W. R. ... ... ... ... ... ... ... | 5 Rodney Place, Clifton. |
| Adams, George ... ... ... ... ... ... ... | 11 Westbourne Place, Clifton. |
| *Adams, John Barfield ... ... ... ... ... ... | 110 Cotham Road, Bristol. |
| Adye, W. John A. ... ... ... ... ... ... ... | Church House, Bradford-on-Avon. |
| Alexander, James, M.D., M.Ch., B.A. Dub. | Bishop's Place, Paignton. |
| *Alford, George Ernest ... ... ... ... ... | 5 Royal Crescent, Weston-super-Mare |
| Alford, Henry ... ... ... ... ... ... ... | 9 Mountlands, Taunton. |
| Alford, Henry J., M.D. Lond. ... ... ... | 2 Marlborough Terrace, Taunton. |
| Arthur, John ... ... ... ... ... ... ... ... | Hilton, Llandaff. |
| *Atchley, George F., M.B. Lond. ... ... ... | Tyndall's Park, Bristol. |
| *Aveline, H. T. S. ... ... ... ... ... ... ... | Asylum, Stapleton, Gloucestershire |
| Baker, A. de W. ... ... ... ... ... ... ... | 2 Lawn Terrace, Dawlish. |
| Ballantyne, J. W., M.D., C.M. Ed. ... ... ... | 24 Melville Street, Edinburgh. |
| Bannatyne, Gilbert A., M.D., C.M. Glasg. ... | 1 Paragon, Bath. |
| *Barclay, W. M. ... ... ... ... ... ... ... | Queen's Road, Clifton. |
| Baretti, T. G. L'Enardi ... ... ... ... ... | 49 Royal York Crescent, Clifton |
| *Baron, Barclay J., M.B., C.M. Ed. ... ... | 16 White Ladies Road, Clifton. |
| *Basset, Walter ... ... ... ... ... ... ... | 24 Stow Hill, Newport, Mon. |
| *Bateman, F. J. B. ... ... ... ... ... ... ... | Alburgh House, Wells, Somersetshire. |
| Batten, Rayner W., M.D. Lond. ... ... ... | 1 Brunswick Square, Gloucester |
| Batten, W. Smith ... ... ... ... ... ... | Curzon House, Calne. |
| *Beddoe, John, M.D. Ed., B.A. Lond., F.R.S. | The Chantry, Bradford-on-Avon. |
| Benham, Harry A., M.D., C.M. Aberd. ... | Asylum, Stapleton, Gloucestershire. |
| Bennett, W. S. ... ... ... ... ... ... ... | Escot, Penzance. |
| *Benson, A. H. ... ... ... ... ... ... ... | Wrington, Somersetshire. |
| Berry, James, M.B., B.S. Lond. ... ... ... | 60 Welbeck Street, London, W. |
| *Blacker, A. E. ... ... ... ... ... ... ... | Richmond Hill Avenue, Clifton. |
| *Blacker, E. ... ... ... ... ... ... ... ... | 12 White Ladies Road, Clifton. |
| *Blagg, Arthur F. ... ... ... ... ... ... | 6 West Mall, Clifton. |
| Blomfield, Arthur G., M.D. Aberd. ... ... | 34 West Southernhay, Exeter. |
| Bloxam, G. E. ... ... ... ... ... ... ... ... | Cambridge House, Lower Weston, Bath. |

### LIST OF SUBSCRIBERS.

| | |
|---|---|
| *Board, Edmund C. ... ... ... ... ... ... ... | 11 Caledonia Place, Clifton. |
| *Boyce, James G. ... ... ... ... ... ... ... | 6 Colston Parade, Bristol. |
| Boys, Arthur Henry ... ... ... ... ... ... ... | Chequer Street, St. Alban's. |
| Brabazon, A. B., M.D. Aberd. ... ... ... ... | 12 Darlington Street, Bath. |
| Bright, J. A. ... ... ... ... ... ... ... ... ... | Glastonbury. |
| *Broom, John, M.D., Ch.D., Obst.D. Brussels | St. Paul's Road, Clifton. |
| Brown, G. Arthur ... ... ... ... ... ... | Tredegar. |
| Brown, William, M.D., C.M. Glasg. ... ... | Fishponds, Gloucestershire. |
| Browne, A. E. Newbury ... ... ... ... ... | Burbage, Wiltshire. |
| Browne, G. Henry ... ... ... ... ... ... | The Hermitage, Brynmawr, Breconshire. |
| Browne, J. Walton, M.D., B.A. Roy. Univ. I. | 10 College Square North, Belfast. |
| Browne, Lennox ... ... ... ... ... ... ... | 15 Mansfield Street, London, W. |
| Burges, R. E., M.D., M.Ch., B.A. Roy. Univ. I. | 3 Egerton Terrace, Hoole Road, Chester. |
| Burroughs, E. F. H. ... ... ... ... ... ... | 3 Elgin Park, Bristol. |
| *Bush, J. Paul ... ... ... ... ... ... ... ... | Clifton Park, Clifton. |
| | |
| *Cameron, John, M.B., C.M. Glasg. ... ... | Ashley Hill, Bristol. |
| *Carr, Alexander ... ... ... ... ... ... ... | Wells Road, Bristol. |
| Carter, R. W., M.D. Calcutta ... ... ... ... | 13 Gloucester Row, Weymouth. |
| *Carter, W. F. ... ... ... ... ... ... ... ... ... | 48 Coronation Road, Bristol. |
| Cave, Edward J., M.D. Lond. ... ... ... ... | Crewkerne. |
| Clapp, W. J. ... ... ... ... ... ... ... ... ... | Earl Street, Penarth. |
| Clark, Thomas ... ... ... ... ... ... ... ... | Blair, Minehead, Somersetshire. |
| *Clarke, John Michell, M.B., M.A. Cantab. | Clifton Road, Clifton. |
| Clay, Challoner ... ... ... ... ... ... ... | Manor House, Fovant. |
| Clay, R. Hogarth, M.D. Ed. ... ... ... ... | 4 Windsor Villas, Plymouth. |
| Close, John B., M.B. Dur. ... ... ... ... | 61 Spring Bank, Hull. |
| Coathupe, Edwin W. ... ... ... ... ... ... | 7 Clifton Park, Clifton. |
| Cockey, Edmund ... ... ... ... ... ... ... ... | West Lodge, Frome. |
| *Coe, R. W. ... ... ... ... ... ... ... ... ... | 7 Pembroke Road, Clifton. |
| Coker, T. V. ... ... ... ... ... ... ... ... ... | 2 Chesterfield Place, Clifton. |
| Cole, Thomas, M.D. Lond. ... ... ... ... | 18 Circus, Bath. |
| *Collins, H. W. ... ... ... ... ... ... ... ... | Wrington, Somersetshire. |
| Colman, Thomas J., M.D. Ed. ... ... ... ... | Elmgrove Road, Bristol. |
| Colmer, Ptolemy S. H., M.D. Dur. ... ... | South Street House, Yeovil. |
| *Cook, Henri, M.B., C.M. Aberd. ... ... ... | 45 Stackpool Road, Bristol. |
| Cooke, A. S. ... ... ... ... ... ... ... ... ... | Badbrook House, Stroud. |
| *Corbould, George G. ... ... ... ... ... | 22 Berkeley Square, Bristol. |
| Cossham, W. R., M.D., C.M. Aberd. ... ... ... | Cirencester. |
| *Cotton, William, M.B., C.M., M.A. Ed. ... | 227 Gloucester Road, Bristol. |
| Cousins, J. Ward, M.D. Lond. ... ... ... | Kent Road, Southsea. |
| Cowan, F. S. ... ... ... ... ... ... ... ... ... | 27 Queen Square, Bath. |
| Cox, William I. ... ... ... ... ... ... ... ... | Hawkesbury-Upton. |
| Craddock, Samuel ... ... ... ... ... ... | South Lyncombe, Bath. |
| Crespi, Alfred J. H. ... ... ... ... ... | Poole Road, Wimborne. |
| *Cross, F. Richardson, M.B. Lond. ... ... | College Road, Clifton. |
| *Crossman, Edward, M.D. Dur. ... ... ... | Hambrook, Gloucestershire. |
| Crouch, C. Percival, M.B. Lond. ... ... ... | 3 Prince's Buildings, Weston-super-Mare. |
| | |
| *Dacre, John ... ... ... ... ... ... ... ... | St. Paul's Road, Clifton. |
| Daniel, T. P. ... ... ... ... ... ... ... ... ... | Beaminster, Dorsetshire. |
| *Davey, James G., M.D. St. And. ... ... ... | 31 Avonmore Road, London, W. |
| *Davies, D. S., M.D. Lond. ... ... ... ... | 60 Oakfield Road, Clifton. |
| Davies, Ebenezer ... ... ... ... ... ... ... | Brunswick House, Swansea. |

### LIST OF SUBSCRIBERS.

| | |
|---|---|
| DAVIES, H. N. ... ... ... ... ... ... ... ... | Porth, Glamorganshire. |
| DAVIS, ROBERT ... ... ... ... ... ... ... ... | Oakleigh, Epsom. |
| DAVIS, THEODORE, M.D. Lond. ... ... ... | Beachcroft, Clevedon. |
| DAVY, HENRY, M.D. Lond.... ... ... ... ... | Southernhay House, Exeter. |
| DAY, PERCY HOWARD ... ... ... ... ... ... | Stalmine, Poulton-le-Fylde. |
| DEAS, P. MAURY, M.B., M.S. Lond. ... ... | Wonford House, Exeter. |
| DENING, EDWIN ... ... ... ... ... ... ... | Manor House, Stow-on-the-Wold. |
| *DEVIS, HARRY F. ... ... ... ... ... ... ... | Wells Road, Bristol. |
| *DEW, HENRY R. ... ... ... ... ... ... ... | 25 Berkeley Square, Bristol. |
| *DOBSON, NELSON C. ... ... ... ... ... ... | 27 Victoria Square, Clifton. |
| DOMVILLE, EDWARD J. ... ... ... ... ... ... | 44 Southernhay, Exeter. |
| *DOWSON, W., M.B., B.C., M.A. Cantab. ... ... | Great George Street, Bristol. |
| DOYNE, ROBERT W. ... ... ... ... ... ... | 70 Woodstock Road, Oxford. |
| DUFFETT, E. D. ... ... ... ... ... ... ... | 5 London Street, Swindon, Wiltshire. |
| DUNBAR, ELIZA L. WALKER, M.D. Zürich ... | 9 Oakfield Road, Clifton. |
| *DUNCAN, WILLIAM ... ... ... ... ... ... ... | Westbury-on-Trym. |
| | |
| EADON, W. F. BAILEY ... ... ... ... ... ... | Hambrook, Gloucestershire. |
| *EAGER, REGINALD, M.D. Lond. ... ... ... | Northwoods, Winterbourne. |
| EDDOWES, CHARLES ... ... ... ... ... ... | Maddington, Devizes. |
| *EDGEWORTH, F. H., M.B., B.C., B.A. Cantab., B.Sc. Lond. ... ... ... ... ... ... ... ... | 1 Richmond Hill, Clifton. |
| EDIS, THOMAS ... ... ... ... ... ... ... ... | 66 Barton Street, Gloucester. |
| EDWARDS, W. T., M.D. Lond. ... ... ... ... | 129 Queen Street, Cardiff. |
| ELDER, WILLIAM ... ... ... ... ... ... ... | 7 Trelawney Road, Bristol. |
| *ELLIOTT, CHRISTOPHER, M.D., B.A. Dub. ... | 88 Pembroke Road, Clifton. |
| ELLIS, T. S. ... ... ... ... ... ... ... ... | 6 Clarence Street, Gloucester. |
| ELLIS, W. Mc.D., M.D. Brussels ... ... ... | 7 Alexander Buildings, Bath. |
| EMBLETON, DENNIS C. ... ... ... ... ... ... | St. Michael's Road, Bournemouth. |
| *ETCHES, W. R. ... ... ... ... ... ... ... | 13 Dowry Square, Clifton. |
| EVANS, J. FENTON, M.B., C.M. Ed. ... ... | 6 Whitehall Yard, London, S.W. |
| EVANS, W. G. ... ... ... ... ... ... ... ... | Beckington. |
| *EWENS, JOHN ... ... ... ... ... ... ... ... | 14 White Ladies Road, Clifton. |
| | |
| FAIRBANKS, W., M.D., C.M. Ed. ... ... ... | Wells, Somersetshire. |
| *FENDICK, R. GEORGE ... ... ... ... ... ... | St. Paul's Road, Clifton. |
| FINCH, THOMAS, M.D. St. And. ... ... ... | Westville, St. Mary Church. |
| FISHER, FRED. B. ... ... ... ... ... ... ... | West Walk, Dorchester. |
| FLEMMING, C. E. S. ... ... ... ... ... ... | Freshford. |
| FORD, JOSEPH ... ... ... ... ... ... ... ... | Wedmore. |
| FORTY, D. H. ... ... ... ... ... ... ... ... | Wotton-under-Edge. |
| FOX, ARTHUR E. W., M.B., C.M. Ed. ... ... | 16 Gay Street, Bath. |
| *FOX, BONVILLE B., M.D., M.A. Oxon. ... ... | Brislington. |
| FOX, CHARLES HENRY, M.D. St. And. ... ... | Brislington. |
| *FOX, E. LONG, M.D. Oxon. ... ... ... ... | Church House, Clifton. |
| FOX, JOHN ALFRED... ... ... ... ... ... ... | Morrab Road, Penzance. |
| FRASER, GRÆME B. ... ... ... ... ... ... | Beach Road, Weston-super-Mare. |
| FREEMAN, HENRY W. ... ... ... ... ... ... | 24 Circus, Bath. |
| FRY, J. BLOUNT ... ... ... ... ... ... ... | Ashley Lodge, Esher. |
| FURSE, EDWIN ... ... ... ... ... ... ... ... | South Molton. |
| *FYFFE, W. JOHNSTONE, M.D., B.A. Dub. ... | 2 Rodney Place, Clifton. |
| | |
| GAMBLE, C. H.... ... ... ... ... ... ... ... | Barnstaple. |
| *GIBBS, ALFRED N. GODBY ... ... ... ... ... | 27 Chandos Road, Bristol. |

### LIST OF SUBSCRIBERS.

| | |
|---|---|
| GILL, JOHN, M.D. Brussels ... ... ... ... ... ... | 31 Apsley Road, Clifton. |
| GLOAG, G. A. ... ... ... ... ... ... ... ... ... ... | 7 College Green, Bristol. |
| GOODDEN, WYNDHAM C. ... ... ... ... ... ... | 14 Berkeley Square, Bristol. |
| GOODRIDGE, HENRY F. A., M.D. Lond. ... | 10 Brock Street, Bath. |
| GORDON, W., M.B., B.C., M.A. Cantab.... ... | Barnfield Lodge, Exeter. |
| GOSS, T. BIDDULPH ... ... ... ... ... ... ... | 1 Circus, Bath. |
| GOULD, A. PEARCE, M.B., M.S. Lond. ... ... | 10 Queen Anne Street, London, W |
| GRACE, ALFRED ... ... ... ... ... ... ... ... | Chipping Sodbury. |
| GRACE, EDWARD MILLS ... ... ... ... ... ... | Thornbury, Gloucestershire. |
| GRACE, HENRY ... ... ... ... ... ... ... ... ... | Kingswood, Bristol. |
| GRACE, W. G. ... ... ... ... ... ... ... ... ... ... | 57 Stapleton Road, Bristol. |
| GRANVILLE, J. MORTIMER, M.D. St. And. ... | 14 Hanover Square, London, W. |
| GRATTE, C. BROOKE ... ... ... ... ... ... ... | 55 Commercial Road, Newport, Mon. |
| GRAY, A. MURRAY ... ... ... ... ... ... ... ... | 17 Market Place, Devizes. |
| GRAY, F. A. ... ... ... ... ... ... ... ... ... | Ottery St. Mary. |
| GREY, T. C. ... ... ... ... ... ... ... ... ... | Swansea Hospital, Swansea. |
| *GRIFFITHS, L. M. ... ... ... ... ... ... ... ... | 9 Gordon Road, Clifton. |
| GROTE, G. W., M.B. Toronto ... ... ... ... | 182 Portway, London, E. |
| GROVES, J., M.B., B.A. Lond. ... ... ... ... | Carisbrooke. |
| GUINNESS, T. A. ... ... ... ... ... ... ... ... | Bampton, Devonshire. |
| | |
| *HAILES, CLEMENTS D. G., M.D., C.M. Ed. ... | 9 King's Parade, Clifton. |
| *HAINES, A. H. ... ... ... ... ... ... ... ... ... | 8 Cotham Road, Bristol. |
| *HALE, EDMUND T. ... ... ... ... ... ... ... ... | The Grange, Chew Magna. |
| HANDFIELD-JONES, MONTAGU, M.D. Lond. ... | 35 Cavendish Square, London, W. |
| HARDWICK, A., M.B. Dur. ... ... ... ... ... | New Quay, Cornwall. |
| HARPER, JOSEPH ... ... ... ... ... ... ... ... | Bear Street, Barnstaple. |
| HARRIS, JOHN H. ... ... ... ... ... ... ... ... | The New Road, Dartmouth. |
| *HARRISON, A. J., M.B. Lond. ... ... ... ... | Guthrie Road, Clifton. |
| HARRISON, JAMES ... ... ... ... ... ... ... ... | 20 Ker Street, Devonport. |
| *HARSANT, W. H. ... ... ... ... ... ... ... ... | 16 Pembroke Road, Clifton. |
| *HAWKINS-AMBLER, G. A. ... ... ... ... ... | 30 Royal Park, Clifton. |
| HAWKINS, CÆSAR F. ... ... ... ... ... ... | North Petherton. |
| HAYES, H. W. McCAULTY ... ... ... ... ... | 1 Brunswick Terrace, Swindon, Wiltshire. |
| HAYDON, EDGAR, M.B., C.M. Glasg. ... ... | The Laurels, Newton Abbot. |
| HAYMAN, ALFRED G. ... ... ... ... ... ... ... | 1 Pembroke Road, Clifton. |
| HEANE, W. C. ... ... ... ... ... ... ... ... ... | Cinderford. |
| HEARDER, G. J., M.D. St. And.... ... ... ... | Joint Counties Asylum, Carmarthen. |
| *HEAVEN, JOHN C. ... ... ... ... ... ... ... ... | 2 Queen Square, Bristol. |
| *HERAPATH, C. K. C. ... ... ... ... ... ... ... | 11 Brunswick Square, Bristol. |
| HERRINGHAM, W. P., M.D., B.A. Oxon. ... ... | 13 Upper Wimpole Street, London, W. |
| *HETLING, H. E. ... ... ... ... ... ... ... ... | Stapleton Road, Bristol. |
| HILL, HEDLEY ... ... ... ... ... ... ... ... ... | 1 Redcliff Hill, Bristol. |
| HILLS, FREDERIC F. ... ... ... ... ... ... ... | 164 Stapleton Road, Bristol. |
| HINTON, JOSEPH ... ... ... ... ... ... ... ... | The Chestnuts, Warminster. |
| HOBLYN, FRANCIS PARKER ... ... ... ... ... | 10 Bladud Buildings, Bath. |
| *HOFFMAN, A. W. W. ... ... ... ... ... ... ... | 25 Gordon Road, Clifton. |
| HOPKINS, H. CULLIFORD ... ... ... ... ... ... | 4 Gay Street, Bath. |
| HORSFALL, JOHN, M.A. Oxon. ... ... ... ... | Bath Road, Bournemouth. |
| HOSPITAL, BRISTOL GENERAL (*Sec.*, W. THWAITES). | |
| HOSPITAL, TAUNTON AND SOMERSET (*House-Surgeon*, BASIL W. HOUSMAN). | |
| HOWELLS, WILLIAM, M.B., C.M. Glasg. ... | Church House, Talgarth, Breconshire. |
| HOWSE, WILLIAM ... ... ... ... ... ... ... ... | 8 London Street, Swindon, Wiltshire. |
| HOWSIN, E. ARTHUR, M.D. St. And. ... ... | Stroud. |
| HUDSON, H. R. ... ... ... ... ... ... ... ... | 182 Commercial Road, Newport, Mon. |

### LIST OF SUBSCRIBERS.

| | |
|---|---|
| Huggard, W. R., M.D., M.Ch., M.A. Roy. Univ. I. | Davos-Platz, Switzerland. |
| Hunt, A. D. | Millbrook House, Chagford. |
| | |
| *Imlay, G. A., M.B., C.M. Aberd. | 1 Apsley Villas, Bristol. |
| Infirmary, Bristol Royal (*Librarian*, Walter J. Hill). | |
| Institution, Liverpool Medical (*Resident Librarian*, William Jones). | |
| Irving, James, M.D. Ed. | 16 Royal York Crescent, Clifton. |
| | |
| Jackson, J. B., M.D., M.Ch. Roy. Univ. I. | New York, U.S.A. |
| Jacobson, W. H. A., M.B., M.C., M.A. Oxon. | 66 Great Cumberland Place, London, W. |
| James, J. Bowen | Woodlands, Aberaman, Aberdare. |
| Jessop, Charles Moore | 98 Sutherland Avenue, London, W. |
| *Johnston, D. G., M.B., C.M. Glasg. | 92 Hampton Road, Bristol. |
| Jones, A. Orlando, M.D., C.M. Aberd. | Harrogate. |
| Jones, E. R. | Cilgynlle-fawr, New Quay, Cardiganshire. |
| Jones, E. S. S. | Cottage Hospital, Dowlais. |
| Jones, Evan | Ty-Mawr, Aberdare. |
| Jones, W. R., M.D., C.M. Glasg. | Senny Bridge, Breconshire. |
| Jones, W. W., M.B., C.M. Ed. | Rhymney, Monmouthshire. |
| | |
| Kemble, A. C. | Glenmalure, Poole. |
| Kempe, Arthur W., M.D. Brussels | 14 Southernhay, Exeter. |
| Kendall, Bernard | Elm Grove, Tenby |
| King, Louis J. | 10 Russel Street, Bath. |
| Kinneir, R. | The Tower House, Malmesbury. |
| Kitson, F. P. | Beaminster, Dorsetshire. |
| | |
| Lace, F. | Royal United Hospital, Bath. |
| Laing, W. A. Gordon, M.D., C.M. Glasg. | Boutport Street, Barnstaple. |
| *Lane, Hugh | 11 Circus, Bath. |
| *Lansdown, F. Poole | 19 White Ladies Road, Clifton. |
| *Lansdown, R. G. P., M.B., B.S. Dur. | 19 White Ladies Road, Clifton. |
| Lauwers, Dr. | Courtrai, Belgium. |
| *Lawrence, A. E. Aust, M.D., C.M. Aberd. | 19 Richmond Hill, Clifton. |
| *Lawrence, Henry | 19 Park Row, Bristol. |
| Lawrie, J. Macpherson, M.D., C.M. Glasg. | Greenhill, Weymouth. |
| Laxton, T. L. | Taunton Road, Bridgwater. |
| Leach, J. Comyns, M.D. Dur., B.Sc. Lond. | Sturminster Newton. |
| Leamon, Michael T. | 1 Bedford Villas, Tavistock. |
| *Lees, E. Leonard, M.D., C.M. Ed. | Redland Road, Bristol. |
| Leigh, W. W. | Treharris, Glamorganshire. |
| Lewellyn, John | Caerphilly. |
| Library, Bristol Museum and | Queen's Road, Bristol. |
| Library, Cheltenham | 5 Royal Crescent, Cheltenham. |
| *Lidderdale, James | Henley-on-Thames. |
| Liddon, William, M.B. Lond. | Church Square, Taunton. |
| Lilley, G. Herbert, M.D. Brussels | H.M. Convict Prison, Portland. |
| Limbery, Thomas | 184 Commercial Road, Newport, Mon. |
| Lister, Sir Joseph, Bart., F.R.S. | 12 Park Crescent, London, N.W. |
| Lloyd, Walter E. | 60 York Road, Bristol. |
| *Logan, Frederic T. B. | Dean Lane, Bristol. |
| *Lowe, T. Pagan | 2 Belmont, Bath. |
| *Lower, N. H. | Olveston, Gloucestershire. |
| Luckham, L. S. | 16 Harcourt Terrace, Salisbury. |

### LIST OF SUBSCRIBERS.

| | |
|---|---|
| Macaskie, James G. ... ... ... ... ... ... | Bamburgh, Northumberland. |
| Macdonald, P. W., M.D., C.M. Aberd. ... | County Asylum, Dorchester. |
| *Maclean, Ewen J., M.D., C.M. Ed. ... ... | Children's Hospital, Bristol. |
| Maclean, J. Campbell, M.B., C.M Ed. ... | Swindon House, Swindon, Wiltshire. |
| Macready, J. ... ... ... ... ... ... ... ... | 51 Queen Anne Street, London, W. |
| *Maddison, W. T., M.D. Lond. ... ... ... ... | 172 Stapleton Road, Bristol. |
| Manning, H. J., B.A. Lond. ... ... ... ... | Laverstock House, Salisbury. |
| Marley, Henry F. ... ... ... ... ... ... | The Nook, Padstow, Cornwall. |
| Marsh, O. E. Bulwer ... ... ... ... ... | Ventnor House, Newport, Mon. |
| Marshall, Arthur L., M.B., B.A. Cantab. ... | 56 Rectory Road, London, N. |
| *Marshall, Henry, M.D. Ed., F.R.S.E. ... | 28 Caledonia Place, Clifton. |
| *Martin, E. F., M.B. Ed. ... ... ... ... ... | Victoria House, Weston-super-Mare. |
| Martin, Theodore ... ... ... ... ... ... | Temple Cloud. |
| Mason, S. B. ... ... ... ... ... ... ... ... ... | 12 Park Terrace, Pontypool. |
| Mason, William ... ... ... ... ... ... ... | Sedgemoor Cottage, St. Austell. |
| Masters, W. Hooper, M.D. St. And. ... ... | Stretton Court, Parkstone. |
| *Matthews, Charles E. ... ... ... ... ... | 31 Pembroke Road, Clifton. |
| Mavrojanni, Dr. ... ... ... ... ... ... ... | Corfu, Greece. |
| *Mayor, T. O. ... ... ... ... ... ... ... ... ... | 40 Apsley Road, Clifton. |
| McNicoll, John ... ... ... ... ... ... ... | High Street, Poole. |
| Meredith, John, M.D. Ed. ... ... ... ... | Wellington, Somersetshire. |
| Metford, J. Seymour ... ... ... ... ... | 34 West Mall, Clifton. |
| Milne, John, M.B., C.M. Aberd. ... ... ... | Hill Avenue, Bristol. |
| Milward, James, M.D. Brussels ... ... ... | 54 Charles Street, Cardiff. |
| Mole, Harold F. ... ... ... ... ... ... ... ... | Royal Infirmary, Bristol. |
| Monckton, William ... ... ... ... ... ... | Portishead. |
| Morgan, Frederick ... ... ... ... ... ... | Uffculme. |
| Morgan, John ... ... ... ... ... ... ... ... | Langport, Somersetshire. |
| *Morton, C. A.... ... ... ... ... ... ... ... | 24 St. Paul's Road, Clifton. |
| Moynan, W. Arthur, M.D., M.Ch. Roy. Univ. I. | Penarth. |
| Munden, Charles ... ... ... ... ... ... ... | Ilminster. |
| Murray, William, M.B., C.M. Ed. ... ... | Staple Hill, Gloucestershire. |
| | |
| Napier, T. W. A., M.D., C.M. Aberd. ... ... | Church Street, Egremont, Cheshire |
| Nash, W. Gifford ... ... ... ... ... ... ... | South Devon Hospital, Plymouth |
| Neale, B. G. ... ... ... ... ... ... ... ... | 23 Fernbank Road, Bristol. |
| *Nevill, W. N., M.D., B.Ch., B.A. Dub. ... | Southville, Bristol. |
| *Newman, Charles ... ... ... ... ... ... ... | 156 Cheltenham Road, Bristol. |
| *Newnham, W. H. C., M.B., M.A. Cantab. ... | St. Paul's Road, Clifton |
| Newstead, James ... ... ... ... ... ... ... | 9 York Place, Clifton. |
| Nicholson, T. D., M.D., C.M. Ed. ... ... | 2 White Ladies Road, Clifton. |
| Norgate, R. H. ... ... ... ... ... ... ... | County Asylum, Powick, Worcestershire. |
| Norman, J. H. ... ... ... ... ... ... ... ... | Goodleigh, Winkleigh, Devonshire. |
| *Norton, J. A., M.D., C.M. Aberd. ... ... ... | 65 Redcliff Hill, Bristol. |
| | |
| *Ogilvy, Alexander, M.D., B.Ch., B.A. Dub. | Eye Hospital, Bristol. |
| Openshaw, E. Hyde ... ... ... ... ... ... | 24 Berkeley Square, Bristol. |
| *Ormerod, Henry ... ... ... ... ... ... ... | Westbury-on-Trym. |
| | |
| Padley, George ... ... ... ... ... ... ... | 2 Northampton Terrace, Swansea. |
| Page, G. Shepley ... ... ... ... ... ... ... | 78 Old Market Street, Bristol. |
| Palmer, F. Craddock, M.D. Brussels ... ... | Woking. |
| *Parker, George, M.D., M.A. Cantab. ... ... | 14 Pembroke Road, Clifton. |

### LIST OF SUBSCRIBERS.

| | |
|---|---|
| Parry, J. H. ... ... ... ... ... ... ... ... | 199 Cheltenham Road, Bristol. |
| Parsons, Francis J. C. ... ... ... ... ... | King Square, Bridgwater. |
| Parsons, Frederick ... ... ... ... ... ... | Common Hill, Frome. |
| Paterson, Donald Rose, M.D., C.M. Ed.... | 18 Windsor Place, Cardiff. |
| Pauli, G. C. P. ... ... ... ... ... ... ... | 240 York Road, Bristol. |
| Pearse, W. ... ... ... ... ... ... ... ... | St. Tudy, Bodmin. |
| *Penny, F. ... ... ... ... ... ... ... ... ... | North Devon Infirmary, Barnstaple. |
| *Penny, W. J. ... ... ... ... ... ... ... ... | Richmond Park Road, Clifton. |
| Pentland, Young J. ... ... ... ... ... ... | 11 Teviot Place, Edinburgh. |
| Peskett, A. W. C., M.B., B.C., M.A. Cantab. | Burnham, Somersetshire. |
| Phelps, W. Harford G., M.D. Aberd. ... | Beachfield, Weston-super-Mare. |
| *Pickering, C. F. ... ... ... ... ... ... ... | 12 Berkeley Square, Bristol. |
| Pippette, Walter... ... ... ... ... ... ... | Dawes Road, London, S.W. |
| Pizey, G. F. P.... ... ... ... ... ... ... ... | 5 Prospect Terrace, Babbacombe. |
| Plaister, W. H. ... ... ... ... ... ... ... | High Road, Tottenham. |
| Pollard, Reginald, M.B. Dur. ... ... ... | Southlands, Torquay. |
| *Pountney, W. E., M.D., C.M. Ed. ... ... | 33a White Ladies Road, Bristol. |
| *Powell, J. J., M.D., Lond. ... ... ... ... | 2 Portland Square, Bristol. |
| Powell, R. Douglas, M.D. Lond.... ... ... | 62 Wimpole Street, London, W. |
| Powell, William, M.B. Lond. ... ... ... | Hill Garden, Torquay. |
| Price, William, M.B. Lond. ... ... ... ... | 40 Park Place, Cardiff. |
| *Prichard, Arthur W.... ... ... ... ... ... | Gordon Road, Clifton. |
| *Prichard, Augustin, M.D. Berlin... ... ... | 4 Chesterfield Place, Clifton. |
| *Prichard, J. E., M.B., B.A. Oxon. ... ... | 243 Cheltenham Road, Bristol. |
| Prichard, R., M.D., C.M. Glasg. ... ... ... | 14 Windsor Place, Cardiff. |
| Prideaux, T. Engledue P. ... ... ... ... | Wellington, Somersetshire. |
| *Pring, Arthur, B.A. Cantab. ... ... ... ... | 14 Meridian Place, Clifton. |
| Prowse, Albert P. ... ... ... ... ... ... | Yennadon, Yelverton, Devonshire. |
| *Prowse, Arthur B., M.D. Lond. ... ... ... | 5 Lansdown Place, Clifton. |
| | |
| Randolph, Charles ... ... ... ... ... ... ... | St. Michael's, Milverton, Somersetshire. |
| *Rattray J. M., M.D., C.M., M.A. Aberd. ... | Frome. |
| Redwood, T. Hall, M.D., C.M., M.A. Dur.... | The Lawn, Rhymney, Monmouthshire. |
| Rendall, John... ... ... ... ... ... ... ... | Forestside, Lymington. |
| Riddell, Andrew W. ... ... ... ... ... ... | St. Mary Bourne. |
| Robathan, G. B. ... ... ... ... ... ... ... | The Grove, Risca. |
| Robertson, Robert, M.D., C.M. Ed. ... ... | Belgrave Road, Ventnor. |
| *Rogers, B. M. H., M.B., B.Ch., B.A. Oxon.... | 11 York Place, Clifton. |
| *Ross, R. A. ... ... ... ... ... ... ... ... | Lodway Villa, Pill. |
| *Rossiter, George F., M.B. Lond. ... ... | Cairo Lodge, Weston-super-Mare. |
| *Roué, W. Barrett, M.D., M.S. Dur. ... ... | 2 Buckingham Place, Clifton. |
| Row, F. Everard ... ... ... ... ... ... ... | 34 Ker Street, Devonport. |
| *Roxburgh, Robert, M.B. Ed.... ... ... ... | 1 Victoria Buildings, Weston-super-Mare. |
| *Rudge, C. King ... ... ... ... ... ... ... | 145 White Ladies Road, Bristol. |
| *Rudge, H. T. ... ... ... ... ... ... ... ... | 5 Colston Parade, Bristol. |
| Ruffer, M. Armand, M.D., B.S., M.A. Oxon. | 19 Iddesleigh Mansions, London, S.W. |
| Rundle, Edmund ... ... ... ... ... ... ... | Royal Cornwall Infirmary, Truro. |
| | |
| Sawyer, J. A. F. ... ... ... ... ... ... ... | Rosslyn, Clevedon. |
| Schleicher, G. ... ... ... ... ... ... ... | Buchhandlung, Odessa, Russia. |
| Scimonelli, Sigra. Cruce ... ... ... ... | 5 Salita dei Cattivi, Via Butera, Palermo. |
| Scott, James, M.B., C.M. Ed.... ... ... ... | 24 Gaolgate Street, Stafford. |
| Scott, Richard J. H. ... ... ... ... ... ... | 28 Circus, Bath. |
| Searle, George C. ... ... ... ... ... ... | Brixham, Devonshire. |

Searle, Richard Burford ... ... ... ... St. Just.
*Semple, H. F.... ... ... ... ... ... ... ... Budleigh Salterton, Devonshire.
Seward, W. J., M.B. Lond. ... ... ... ... Asylum, Colney Hatch, London, N.
*Shaw, J. E., M.B., C.M. Ed. ... ... ... ... 23 Caledonia Place, Clifton.
Shaw, Jesse, M.D.... ... ... ... ... ... ... Fort Beaufort, Cape Colony.
Sheen, A., M.D. St. And. ... ... ... ... ... 23 Newport Road, Cardiff.
*Sheppard, E. J. ... ... ... ... ... ... ... 16 Park Place, Clifton.
*Shorland, Walter E. ... ... ... ... ... 189 Cheltenham Road, Bristol.
Shortridge, Thomas Wood, M.D. Brussels Honiton.
*Sinclair, David, M.B., C.M. Aberd. ... ... 8 Osborne Road, Clifton.
Sincock, J. Bain ... ... ... ... ... ... ... Friarn Street, Bridgwater.
Skeate, Edwin ... ... ... ... ... ... ... 16 Paragon, Bath.
*Skelton, Henry, M.D. St. And. ... ... ... Downend, Gloucestershire.
*Skerritt, E. Markham, M.D., B.S., B.A. Lond. Richmond Hill, Clifton.
*Skinner, Stephen, M.B., C.M. Aberd. ... Tranent Lawn, Clevedon.
Smith, Frederick A. A., M.D., C.M. Glasg. Portland House, Cheltenham.
*Smith, G. Munro ... ... ... ... ... ... ... 28 Alma Road, Clifton.
*Smith, J. Greig, M.B., C.M., M.A. Ab., F.R.S.E. 16 Victoria Square, Clifton.
Smith, Noble ... ... ... ... ... ... ... ... 24 Queen Anne Street, London, W.
*Smith, R. Shingleton, M.D., B.Sc. Lond. Clifton Park, Clifton.
Smith, Samuel ... ... ... ... ... ... ... 7 Kingsdown Parade, Bristol.
Smith, W. Alexander, M.B., M.A. Oxon. ... Newport, Essex.
Snell, Simeon ... ... ... ... ... ... ... 17 Eyre Street, Sheffield.
Society, Cardiff Medical (*Hon. Sec.*, Dr. D. R. Paterson).
Society, Royal Medical and Chirurgical 53 Berners Street, London, W.
*Solly, Reginald V., M.B., B.S. Lond. ... General Hospital, Bristol.
Somer, James ... ... ... ... ... ... ... ... Broadclyst.
*Spencer, W. H., M.D., M.A. Cantab. ... ... 11 Warrior Square, St. Leonard's-on-Sea.
Spender, John K., M.D. Lond. ... ... ... 17 Circus, Bath.
Sproule, A. Arnold ... ... ... ... ... ... 63 Stackpool Road, Bristol.
Stamp, W. D. ... ... ... ... ... ... ... ... Ridgeway House, Plympton.
Stansfeld, G. M. ... ... ... ... ... ... ... 19 Victoria Square, Clifton.
Statham, R. Whiteside ... ... ... ... ... Cheddar, Somersetshire
*Steele, Charles, M.D. Dur. ... ... ... ... 17 Richmond Hill, Clifton.
Steele-Perkins, E. B. ... ... ... ... ... 1 Hill's Buildings, Exeter.
*Stevens, W. H. ... ... ... ... ... ... ... Gloucester Road, Bristol.
Stoddart, F. Wallis ... ... ... ... ... ... 1 Park Street, Bristol.
Storry, Frederick W. ... ... ... ... ... Lansdown, Stroud.
*Swain, James, M.D., M.S. Lond. ... ... ... Royal Infirmary, Bristol.
*Swayne, J. G., M.D. Lond. ... ... ... ... 74 Pembroke Road, Clifton.
*Swayne, S. H.... ... ... ... ... ... ... ... 129 Pembroke Road, Clifton.
*Swayne, W. Carless, M.B. Lond.... ... ... St. Paul's Road, Clifton.
Sydenham, George F. ... ... ... ... ... Dulverton, Somersetshire.
Symonds, H. P. ... ... ... ... ... ... ... 35 Beaumont Street, Oxford.
Symons, John ... ... ... ... ... ... ... ... Buriton House, Penzance.

Tait, Lawson ... ... ... ... ... ... ... ... 7 The Crescent, Birmingham
Taylor, C. Bell, M.D. Edin. ... ... ... ... 9 Park Row, Nottingham.
*Taylor, James... ... ... ... ... ... ... ... 36 Alma Road, Clifton.
Taylor, Thomas Henry ... ... ... ... ... Thornbury, Gloucestershire.
Thomas, A. Garrod, M.D., C.M. Ed. ... ... Clytha Park, Newport, Mon.
Thomas, John T. ... ... ... ... ... ... ... Chepstow Road, Newport, Mon.
Thompson, J. Tatham, M.B., C.M. Ed. ... 23 Charles Street, Cardiff.
Thomson, Eustace B., M.D. C.M. Glasg.... Albany Place, Plymouth.

### LIST OF SUBSCRIBERS.

| | |
|---|---|
| THOMSON, WILLIAM, M.D., C.M. Ed. ... ... | Mustapha Superieur, Algiers. |
| *TIVY, W. J. ... ... ... ... ... ... ... ... | 8 Lansdown Place, Clifton. |
| TOMKINS, HARDING H.... ... ... ... ... ... | 36 Ventnor Villas, Brighton. |
| TORBOCK, W. H., M.D. Pennsylvania ... ... | Polruan, Cornwall. |
| TOSSWILL, LOUIS H., M.B., B.A. Cantab. ... | 28 West Southernhay, Exeter. |
| TROTTER, LESLIE B., M.D., C.M. Ed. ... ... | Coleford, Gloucestershire. |
| TWINING, A. H. ... ... ... ... ... ... ... | The Knoll, Salcombe. |
| TYLEY, R. P., M.D. St. And. ... ... ... ... | Wedmore. |
| | |
| VACHELL, HERBERT R., M.D., C.M. Aberd.... | 15 Newport Road, Cardiff. |
| VERNON, EDWARD ... ... ... ... ... ... ... | Kingston, Yeovil. |
| | |
| WADE, A. LAW, M.D., B.A. Dub. ... ... ... | County Asylum, Wells, Somersetshire. |
| WADE, CHARLES H., M.A. Oxon. ... ... ... | Chudleigh. |
| WADE, REGINALD ... ... ... ... ... ... ... | Highbridge, Somersetshire. |
| *WALDO, HENRY, M.D., C.M. Aberd. ... ... | 19 Pembroke Road, Clifton. |
| *WALKER, CYRIL H., M.B., B.A. Cantab.... ... | St. Paul's Road, Ciflton. |
| WALLACE, REV. CANON, M.A. Oxon. ... ... | 3 Harley Place, Clifton. |
| WALTER, WILLIAM HENRY, M.D. Brussels ... | Gregory Boulevard, Nottingham. |
| WARD, J. L. W. ... ... ... ... ... ... ... ... | Victoria Street, Merthyr Tydvil. |
| *WATHEN, J. HANCOCKE ... ... ... ... ... ... | 16 York Place, Clifton. |
| WATSON, J. ADAM ... ... ... ... ... ... ... ... | 39 Dennington Park, London, N.W. |
| WATTERS, GEORGE T. B., M.D., C.M. Ed. ... | Stonehouse, Gloucestershire. |
| WAUGH, ALEXANDER ... ... ... ... ... ... | Midsomer Norton. |
| WEATHERLY, LIONEL A., M.D., C.M. Aberd. | Bailbrook House, Bath. |
| WEBBER, WILLIAM W.... ... ... ... ... ... | Crewkerne. |
| WEBSTER, NORMAN P. ... ... ... ... ... ... | George Place, Guernsey. |
| *WEBSTER, THOMAS ... ... ... ... ... ... | Redland Hill, Bristol. |
| *WEDMORE, C. ERNEST, M.B., M.A. Cantab. | 11 Richmond Hill, Clifton. |
| WETHERED, FRANK J., M.D. Lond. ... ... | 34 Queen Anne Street, London, W. |
| *WHICHER, A. H. ... ... ... ... ... ... ... ... | Midsomer Norton. |
| WHITFELD, W. CLARKE ... ... ... ... ... ... | Baggally Street, Hereford. |
| WHYTE, ANDREW, M.D., C.M. Aberd. ... ... | Honddû House, Brecon. |
| WICKHAM, W. ... ... ... ... ... ... ... ... ... | Hill House, Tetbury. |
| WIGAN, C. A., M.D. Dur. ... ... ... ... ... | Portishead. |
| WIGMORE, J., M.D. Roy. Univ. I. ... ... ... | Twerton-on-Avon. |
| *WILDING, JAMES, M.D. Dur. ... ... ... ... | Sydenham Road, Bristol. |
| WILLCOX, R. LEWIS ... ... ... ... ... ... | Warminster. |
| WILLETT, GEORGE G. D. ... ... ... ... ... | Keynsham. |
| WILLIAMS, CHARLES ... ... ... ... ... ... | Howard Gardens, Cardiff. |
| WILLIAMS, D. J. ... ... ... ... ... ... ... ... | Greenfield House, Llanelly. |
| WILLIAMS, H. EGERTON ... ... ... ... ... | The Mount, Newport, Mon. |
| *WILLIAMS, LIONEL H. ... ... ... ... ... ... | Thornbury, Gloucestershire. |
| *WILLIAMS, P. WATSON, M.B. Lond. ... ... | 14 Westbourne Place, Clifton. |
| WILLIAMS, PETER ... ... ... ... ... ... ... | Ferryside, Carmarthenshire. |
| WILSON, CLAUDE, M.D., C.M. Ed. ... ... ... | Church Road, Tunbridge Wells. |
| *WINDSOR-AUBREY, H. W. ... ... ... ... ... | Coronation Road, Bristol. |
| WINTERBOTHAM, L. ... ... ... ... ... ... | Bays Hill, Cheltenham. |
| WINTLE, COLSTON ... ... ... ... ... ... ... | Belgrave Road, Bristol. |
| WOOLLCOMBE, W. L. ... ... ... ... ... ... | 16 Lockyer Street, Plymouth. |
| | |
| YELF, LEONARD K., M.D. St. And. ... ... ... | Moreton-in-Marsh. |

### The Library of the Bristol Medico-Chirurgical Society.

*The following donations have been received since the publication of the List in December:*

*February 29th*, 1892.

| | |
|---|---|
| J. Michell Clarke, M.B. (1) ... ... ... ... | 22 volumes. |
| W. Duncan. (2) ... ... ... ... ... ... ... ... | 2 ,, |
| L. M. Griffiths. (3) ... ... ... ... ... ... ... | 5 ,, |
| Clements D. G. Hailes, M.D. (4) ... ... ... | 1 volume. |
| W. H. Harsant. (5) ... ... ... ... ... ... ... | 39 volumes. |
| J. E. Shaw, M.B. (6) ... ... ... ... ... | 2 ,, |
| R. Shingleton Smith, M.D. (7) ... ... ... | 1 volume. |
| J. G. Swayne, M.D. (8) ... ... ... ... ... ... | 1 ,, |

Framed pictures have been presented by Miss Amy Blew, Mr. Nelson Dobson, and Dr. Shingleton Smith.

### THIRD LIST OF BOOKS.

The figures in brackets refer to the figures after the names of the donors, and show by whom the volumes were presented. The books to which no such figures are attached have either been bought from the Library Fund or received through the *Journal.*

---

**Aitken, W.** ... *The Science and Practice of Medicine.* (1) Vol. II. 3rd Ed. 1864
*Aneurism, Observations on* (Tr. and Ed. by J. E. Erichsen) ... (5) 1844
**Aretæus** (The Cappadocian). *Extant Works*—Greek and English. (Ed. and Tr. by F. Adams) ... ... ... (5) 1856
**Auld, A. G.** ... *The Pathology of Bronchial Affections and Pneumonia* 1891
**Aulde, J. V. Shoemaker and J.** *Materia Medica, Pharmacology, and Therapeutics* ... ... ... ... ... (6) Vol. I. 1889

**Ballance and W. Edmunds, C. A.** *Ligation in Continuity* ... ... ... 1891
**Barr, J.** ... ... *The Treatment of Typhoid Fever* ... ... ... ... 1892
**Belcher, Rev. Dr.** *The Miracles of Healing* ... ... ... 2nd Ed. [1890]
**Bigg, H. H.** ... *On Deformities.* Part II. The Spine and Upper Extremities ... ... ... ... ... ... ... (1) 1862
**Bramwell, B.** ... *Atlas of Clinical Medicine.* Vol. I., Part III. ... 1891
**Bristowe, J. S.** ... *The Theory and Practice of Medicine.* (1) 2nd Ed. 1878
**Buret, F.** ... ... *Syphilis To-day and Among the Ancients.* (Tr. by A. H. Ohmann-Dumesnil). Vol. I. ... ... 1891

**Chelius, J. M.** ... *System of Surgery.* (Tr. by J. F. South.) 2 vols. (1) 1847
**Coltman, R., Jr.** ... *The Chinese—Medical, Political, and Social* ... ... 1891

**Dale, W.** ... ... *Inherited Consumption* ... ... ... ... ... ... 1891
**Davis, N. S., Jr.** ... *Consumption* ... ... ... ... ... ... ... ... ... 1891
**Dupuytren, Baron** *Diseases and Injuries of Bones.* (Tr. and Ed. by F. le Gros Clark) ... ... ... ... ... ... (5) 1847
,, *Lesions of Vascular System.* (Tr. and Ed. by F. le Gros Clark) ... ... ... ... ... ... (5) 1854

**Edmunds, C. A. Ballance and W.** *Ligation in Continuity* ... ... ... 1891
**Erichsen, J.** ... *Science & Art of Surgery* (1) 2nd Ed. 1857 (1) 4th Ed. 1864

**Feuchtersleben, Baron.** *Medical Psychology.* (Tr. by H. E. Lloyd and Ed. by B. G. Babington) ... ... ... (5) 1847
**Foster, M.** ... ... *Practical Physiology.* (Assisted by J. N. Langley). (1) 4th Ed. 1880

**Foster, M.** ... ... *Text-book of Physiology.* Parts III., IV. 5th Ed. Revised ... ... ... ... ... ... ... ... 1890-91
**Fraser, A.** ... ... *A Guide to Operations on the Brain* ... ... ... 1890

**Gotch and V. Horsley, F.** *On the Mammalian Nervous System* ... ... 1891

**Gruber, J.** ... ... *Diseases of the Ear.* (Tr. and Ed. by E. Law and C. Jewell) ... ... ... ... ... ... ... 1890

**Hare, H. A.** ... *Epilepsy: its Pathology and Treatment* ... ... (6) 1890
**Harris and D. Power, V.** *Physiological Laboratory.* (1) 2nd Ed. 1882
(1) 3rd Ed. 1884
**Hartridge, G.** ... *The Ophthalmoscope* ... ... ... ... ... ... ... ... 1891
**Harvey, W.** ... *Works.* (Tr. by R. Willis) ... ... ... ... (5) 1847
**Hasse, C. E.** ... *Pathological Anatomy.* (Tr. and Ed. by W. E. Swaine) ... ... ... ... ... ... ... ... (5) 1846
**Herman, G. E.** ... *First Lines in Midwifery* ... ... ... ... ... ... 1891
**Hewson, W.** ... *Works.* (Ed. by G. Gulliver) ... ... ... (5) 1846
**Hillis, J. D.** ... *Leprosy in British Guiana* ... ... ... ... (2) 1881
**Hippocrates (Cos)** *Genuine Works.* (Tr. and Ed. by F. Adams.) 2 vols. ... ... ... ... .. ... ... ... (5) 1849
**Horsley, F. Gotch and V.** *On the Mammalian Nervous System.* ... ... 1891
**Huidekoper, R. S.** *Age of the Domestic Animals* ... ... ... ... ... 1891

*Infectious and Contagious Diseases in Schools, A Code of Rules for the Prevention of* ... ... ... ... ... ... 3rd Ed. 1891
*Influenza, Annals of* (Ed. by T. Thompson) ... ... ... ... ... (5) 1852

**Jessop, C. M.** ... *Saturn's Kingdom* ... ... ... ... ... ... ... ... 1891

**Johnson, E.** ... *Results of Hydropathy* ... ... ... ... ... ... (1) 1846

**Kingsbury, G. C.** *The Practice of Hypnotic Suggestion* ... ... ... 1891
**Kirkes, W. S.** ... *Hand-book of Physiology.* (1) 2nd Ed. (Assisted by J. Paget) 1851. (1) 5th Ed. 1863
**Kölliker, A.** ... *Manual of Human Histology.* (Tr. and Ed. by G. Busk and T. Huxley.) 2 vols. ... ... 1853-54
**Küchenmeister, F.** *Manual of Parasites.* 2nd Ed. (Tr. by E. Lankester.) 2 vols. ... ... ... ... ... (5) 1857

**Louis, P. C. A.** ... *Researches on Phthisis.* 2nd Ed. (Tr. by W. H. Walshe) ... ... ... ... ... ... ... ... (5) 1844

**Maclaren, P. H.** ... *Atlas of Venereal Diseases* ... ... ... ... (4) 1886
**Maclise, J.** ... ... *Surgical Anatomy* ... ... ... ... (2) 2nd Ed. 1856
**Martindale, W.** ... *Coca and Cocaine* ... ... ... ... ... 2nd Ed. 1892
**M'Bride, P.** ... *Diseases of the Throat, Nose, and Ear* ... ... ... 1892
**Moore, J. W.** ... *Eruptive and Continued Fevers* ... ... ... ... ... 1892

**Nayler, G.** ... ... *Diseases of the Skin* ... ... ... ... ... ... (1) 1866

**Oesterlen, F.** ... *Medical Logic.* (Tr. and Ed. by G. Whitley.) (5) 1855

**Paulus** (Ægineta) *Seven Books.* (Tr. by F. Adams.) 3 vols. (5) 1844–47
**Politzer, A** ... *The Human Ear.* (Tr. by G. Stone) ... . ... 1892
**Power, V. Harris and D.** *Physiological Laboratory.* (1) 2nd Ed. 1882
(1) 3rd Ed. 1884
**Prochaska, J. A Unzer and G.** *On the Nervous System.* (Tr. and Ed. by T. Laycock) ... ... ... ... ... ... (5) 1851
**Puschmann, T.** ... *History of Medical Education.* (Tr. by E. H. Hare) 1891
**Pye, W.** ... ... *Surgical Handicraft.* 3rd Ed. (Ed. by T. H. R. Crowle) ... ... ... ... ... ... ... ... 1891

**Ramsbotham, F. H.** *Obstetric Medicine and Surgery.* (1) 2nd Ed. 1844
**Rhazes, —**... .. *On the Small-pox and Measles.* (Tr. by W. A. Greenhill) ... ... ... ... ... ... ... (5) 1848
**Roberts, W.** ... *On Urinary and Renal Diseases* ... (1) 2nd Ed. 1872
**Rokitansky, C.** ... *Pathological Anatomy.* 4 vols. (5) Vol. I. (Tr. by W. E. Swaine) 1854; (5) Vol. II. (Tr. by E. Sieveking) 1849; (5) Vol. III. (Tr. by C. H. Moore) 1850; (5) Vol. IV. (Tr. by G. E. Day) ... ... ... ... ... ... .. ... 1852
**Romberg, M. H.**... *Diseases of the Nervous System.* (Tr. and Ed. by E. H. Sieveking.) 2 vols.... ... ... ... (5) 1853
**Rose, W** . ... ... *Harelip and Cleft Palate* ... ... ... ... ... ... 1891

**Schleiden, T. Schwann and M. J.** *Researches.* (Tr. by H. Smith.) (5) 1847
**Schwann and M. J. Schleiden, T.** *Researches.* (Tr. by H. Smith.) (5) 1847
**Shaw, J.** ... ... *Epitome of Mental Diseases* ... ... ... ... ... ... 1892
**Shoemaker and J. Aulde, J. V.** *Materia Medica, Pharmacology, and Therapeutics* ... ... ... ... ... (6) Vol. I. 1889
**Siebold, C. T. v.** ... *On Worms.* (Tr. by T. H. Huxley) ... ... (5) 1857
**Simon, J. F.** ... *Chemistry.* (Tr. and Ed. by G. E. Day.) 2 vols. (5) 1845-46
**Sisley, R.** ... ... *Epidemic Influenza* ... ... ... ... ... .. ... 1891
,, ... ... *A Study of Influenza and the Laws of England concerning Infectious Diseases.* .. ... [2nd Ed.] 1892
**Steven, J. L.** ... *Mediastinal Tumours* ... ... ... ... ... ... ... 1892
*Surgery, Memoirs of the Academy of* (Tr. and Ed. by D. Ottley.) (5) 1848
**Sutton, J. B.** ... *Surgical Diseases of the Ovaries and Fallopian Tubes* 1891
**Swayne, J. G.** ... *Obstetric Aphorisms* ... ... ... (8) 9th Ed. 1888
**Sydenham, T.** ... *Opera Omnia.* (Ed. by W. A. Greenhill.) (5) 1844
,, ... *Works.* (Tr. by R. G. Latham.) 2 vols. (5) 1848–50

**Tanner, T. H.** ... *The Practice of Medicine.* 2 vols. (1) 6th Ed. 1869
**Thomson, T.** ... *Animal Chemistry* ... ... ... ... ... ... (1) 1843

**Treves, F.** ... ... *A Manual of Operative Surgery.* 2 vols. ... ... 1891
**Tuckey, C. L.** ... *Pyscho-Therapeutics* ... ... ... ... ... 3rd Ed. 1891

**Unzer and G. Prochaska, J. A.** *On the Nervous System.* (Tr. and Ed. by T. Laycock) ... ... ... ... ... ... (5) 1851

*Vaccination Commission.* 3rd Report ... ... ... ... ... ... (5) 1890
**Velpeau, A.** ... *Diseases of the Breast.* (Tr. by M. Henry.) (5) 1856

**Wedl, C.** ... ... *Pathological Histology.* (Tr. and Ed. by G. Busk.) (5) 1855
*Women, Diseases Peculiar to* (Ed. by F. Churchill) ... ... ... (5) 1849

---

### TRANSACTIONS, REPORTS, JOURNALS, &c.

*The List of current Periodicals to be seen in the Library is given in another part of this Number.*

Bristol Medico-Chirurgical Journal. Vol. IX. ... ... ... ... 1891
British Journal of Dermatology, The Vol. III. ... ... ... ... 1891
British Medical Journal, The Vol. II. for 1891 ... ... ... .. 1891
British Medical Journal, Supplement to the ... ... 1891. II. ... 1891
Glasgow Medical Journal, The Vol. XXXVI.... ... ... ... ... 1891
Hospital, The Vol. X. ... ... ... ... ... ... ... ... ... ... 1891
Hospitals—
Johns Hopkins Hospital Reports. Vol. II., Nos. 7, 8, 9 ... 1891
Middlesex Hospital. Registrars' Reports... ... ... ... ... 1890
Journal of Cutaneous and Genito-Urinary Diseases. Vol. IX. ... 1891
Journal of Physiology, The ... ... ... ... ... (7) Vol. XI. 1890
Lancet, The Vol. II. for 1891 ... ... ... ... ... ... ... ... 1891
Medical Directory, The... ... ... ... ... ... ... ... ... ... 1892
Medical Record, The Vol. XL. ... ... ... ... ... ... ... ... 1891
New York Medical Journal, The Vol. LIV. ... ... ... ... ... 1891
Progrès médical, Le ... ... (1) Tomes VII. and VIII., 1888; (1) Tomes IX. and X., 1889; (1) Tomes XI. and XII. 1890
Retrospect of Medicine, Braithwaite's (3) Vols. LXV.—LXIX. 1872–74
Therapeutic Gazette, The Vol. XV.... ... ... ... ... ... ... 1891
Union médicale, L' 3rd Series. Tome LII. ... ... ... ... ... 1891
Year-book of Treatment, The ... ... ... ... ... ... ... ... 1892

In Vol. ix., p. 318, line 4 from top, for 1821 read 1891.

### Bristol Medico-Chirurgical Society.

### President.

F. R. CROSS, M.B. Lond.

### President-Elect.

E. MARKHAM SKERRITT, M.D., B.S., B.A. Lond.

### Honorary Secretary.

G. MUNRO SMITH.

### Committee.

*Past Presidents.*
R. W. COE.
AUGUSTIN PRICHARD, M.D. Berlin.
J. G. SWAYNE, M.D. Lond.
E. LONG FOX, M.D. Oxon.
JAMES G. DAVEY, M.D. St. And.
W. JOHNSTONE FYFFE, M.D. Dub.
W. H. SPENCER, M.D., M.A. Cantab.
F. POOLE LANSDOWN.
L. M. GRIFFITHS.
R. SHINGLETON SMITH, M.D., B.Sc. Lond.
NELSON C. DOBSON.
S. H. SWAYNE.

J. MICHELL CLARKE, M.B., M.A. Cantab.
A. J. HARRISON, M.B. Lond.
W. H. HARSANT.
ARTHUR W. PRICHARD.
J. E. SHAW, M.B., C.M. Ed.
J. GREIG SMITH, M.B., C.M., M.A. Aberd., F.R.S.E.

Medical Men wishing to join the Society are requested to communicate with the Honorary Secretary, G. MUNRO SMITH, 28 Alma Road, Clifton, Bristol. The Annual Subscription is 10/6.

---

*Extracts from Journal By-laws.*

4.—The *Journal* shall be delivered free to Subscribers and also to Members of the Society whose subscriptions are not in arrear.

6.—The Annual Subscription to the *Journal* for those who pay before the 1st of July shall be 5/-, post free, or 6/- if paid after that date.

---

Communications intended for insertion in the *Journal* should be sent to the Editor, Dr. SHINGLETON SMITH, Deepholm, Clifton Park, Clifton, Bristol.

Books for Review, Exchange Journals, and communications referring to the delivery of the *Journal* should be sent to the Assistant-Editor, L. M. GRIFFITHS, 9 Gordon Road, Clifton, Bristol, to whom Subscriptions are to be paid *in advance.*

Authors requiring Reprints of their Papers should communicate with the Publisher.

Cases for Binding Vols. I.–IX. are now ready, and may be obtained, price 7*d.* each (postage 2*d.* extra), upon application to J. W. ARROWSMITH, Quay Street, Bristol; or the *Journal* will be bound, including Case, for 1/3 per volume.

### PUBLICATIONS RECEIVED.

From The American Medical Press Company, Limited, Philadelphia:
*Apparatus for Collecting Water for Bacteriological Examination.* By Samuel G. Dixon, M.D. Reprint.
From 190 Baymiller Street, Cincinnatti:
*The Ohio Medical Journal.* January, 1892.
From Beaumont & Co., London:
*Hygiene.* January, 1892.
From Cassell & Company Limited, London:
*Surgical Diseases of the Ovaries and Fallopian Tubes.* By J. Bland Sutton. 1891.
From J. & A. Churchill, London:
*A Code of Rules for the Prevention of Infectious and Contagious Diseases in Schools.* Third and Revised Edition. 1891.
*The Ophthalmoscope.* By Gustavus Hartridge. 1891.
From T. & A. Constable, Edinburgh:
*Atlas of Clinical Medicine.* By Byrom Bramwell, M.D. Vol. I. Part III. 1891.
From Daix Frères, Clermont:
*Revue Internationale de Rhinologie, Otologie and Laryngologie.* 20 Fevrier. 1892.
From F. A. Davis, Philadelphia:
*The Chinese: Medical, Political, and Social.* By Robert Coltman, jun., M.D. 1891.
*Consumption.* By N. S. Davis, jun., M.D. 1891.
*Syphilis To-Day and Among the Ancients.* Vol. I. *Syphilis in Ancient and Prehistoric Times.* By Dr. F. Buret. Translated by A. H. Ohmann-Durmesnil, M.D. 1891.
From Prof. Samuel G. Dixon, M.D. Philadelphia:
*Annual Addrss before the State Board of Health of Pennsylvania.* By Prof. Samuel G. Dixon, M.D. Read May 15, 1891.
From Fannin & Co., Dublin:
*Eruptive and Continued Fevers.* By John William Moore, M.D. 1892.
From T. Fletcher & Co., Warrington:
*A New Patent Calendar for* 1892.
From John Hagyard, Scarborough:
*Scarborough Rural Sanitary Authority. Annual Report for* 1891. By R. Cuff, M.B.
From Hugh Hopkins, Glasgow:
*Inaugural Address to the Physiology Class in Anderson's College. Session* 1891—92. By D. Campbell Black, M.D. 1891.
From The Johns Hopkins Press, Baltimore:
*The Johns Hopkins Hospital Reports.* Vol. II. Nos. 7—8—9. 1891.
From H. K. Lewis, London:
*Harelip and Cleft Palate.* By William Rose, M.B., B.S. 1891.
*The Middlesex Hospital. Reports of the Medical, Surgical, and Pathological Registrars for the Year* 1890. 1891.
*The British Journal of Dermatology.* January—March, 1892.
*Mediastinal Tumours.* By John Lindsay Steven, M.D. 1892.
*Coca and Cocaine.* By William Martindale. Second Edition. 1892.
From Longmans, Green & Co., London:
*A Study of Influenza and the Laws of England concerning Infectious Diseases.* By Richard Sisley, M.D. [Second Edition.] 1892.
From Macmillan & Co., London:
*Ligation in Continuity.* By Charles A. Ballance, M.B., M.S., and Walter Edmunds, M.C. 1891.
From Kegan Paul, Trench, Trübner & Co. Limited, London:
*Saturn's Kingdom.* By Charles Moore Jessop. 1891.
*On the Mammalian Nervous System.* By Francis Gotch and Victor Horsley, F.R.S. 1891.
From Young J. Pentland, Edinburgh:
*Diseases of the Nose, Throat, and Ear.* By P. McBride, M.D. 1892.
From John Morgan Richards, London:
*Medical Reprints.* January—March, 1892.
From Sacklins & Co., London:
*Medical Press and Circular.* February 24, 1892.
From W. R. Saunders, Philadelphia:
*The Climatologist.* February, 1892.
From Wiebeck y Turtl, Buenos Aires:
*Anales del Departmento Nacional de Higiene.* Octobre, 1891.
From John Wright & Co., Bristol:
*Pye's Surgical Handicraft.* Third Edition. Revised and Edited by T. H. R. Crowle. 1891.
*Epitome of Mental Diseases.* By James Shaw, M.D. 1892.

### Bristol Medico-Chirurgical Journal.

### MARCH, 1892.

### CONTENTS.

ORIGINAL PAPERS: Page

A Recent Outbreak of Rötheln. By WILLIAM JOHNSTONE FYFFE, M.D. 1

On a Rare Form of Spina Bifida (Syringo-Myelocele). By CHARLES A. MORTON, F.R.C.S. Eng. ... ... ... ... ... ... ... ... ... ... 14

The Quantitative Estimation of the Knee-Jerk. By EWEN J. MACLEAN, M.D., C.M. Edin. ... ... ... ... ... ... ... ... ... ... ... 20

PROGRESS OF THE MEDICAL SCIENCES ... ... ... ... ... ... ... ... 26

REVIEWS OF BOOKS ... ... ... ... ... ... ... ... ... ... ... ... ... 45

NOTES ON PREPARATIONS FOR THE SICK ... ... ... ... ... ... ... ... 57

MEETINGS ... ... ... ... ... ... ... ... ... ... ... ... ... ... ... 59

THE LIBRARY OF THE BRISTOL MEDICO-CHIRURGICAL SOCIETY ... ... ... 62

OBITUARY ... ... ... ... ... ... ... ... ... ... ... ... ... ... ... 67

INTERNATIONAL CONGRESS OF GYNÆCOLOGY AND OBSTETRICS ... ... ... 68

ALVARENGA PRIZE OF THE COLLEGE OF PHYSICIANS OF PHILADELPHIA ... 69

SCRAPS ... ... ... ... ... ... ... ... ... ... ... ... ... ... ... 69

EDITORIAL NOTE ... ... ... ... ... ... ... ... ... ... ... ... ... 72

---

*Cases for binding, price 7d. each, may be had of the Publisher,*
J. W. ARROWSMITH, *Quay Street, Bristol.*

### Exchange-List.

### AMERICA.

### ARGENTINE REPUBLIC.

*Monthly.*

*Anales del Circulo Médico Argentino.* Buenos Ayres: M. Biedma.

### CANADA.

*Monthly.*

*The Montreal Medical Journal.* Montreal: Gazette Printing Company.

### MEXICO.

*Twice a month.*

*Gaceta Médica.* Mexico: Avenida 2 Oriente, 726.

### PERU.

*Monthly.*

*La Crónica Médica.* Lima: Carlos Prince.

### UNITED STATES.

*Weekly.*

*Medical Review.* St. Louis: J. H. Chambers & Co.

*The Cincinnati Lancet-Clinic.* Cincinnati: 199 West 7th Street.

*The Journal of the American Medical Association.* Chicago: 68 Wabash Avenue.

*The Medical and Surgical Reporter.* Philadelphia: N.E. Cor. 13th and Walnut Streets.

*The Medical News.* Philadelphia: Lea Brothers & Co.

*The Medical Record.* New York: William Wood & Co.

*The New York Medical Journal.* New York: D. Appleton & Co.

*Fortnightly.*

*The American Practitioner & News.* Louisville: John P. Morton and Company.

*Twice a month.*

*The Medical Age.* Detroit: George S. Davis.

*Monthly.*

*American Journal of Obstetrics.* New York: William Wood & Co.

*Annals of Surgery.* St. Louis: J. H. Chambers & Co.

*Index Medicus.* Detroit: George S. Davis.

*Journal of Cutaneous and Genito-Urinary Diseases.* New York: D. Appleton & Co.

*The American Journal of Ophthalmology.* St. Louis: J. H. Chambers & Co.

*The American Journal of the Medical Sciences.* Philadelphia: Lea Brothers & Co.

*The Journal of Nervous and Mental Disease.* New York: 25 West 45th Street.

*The Epitome of Medicine.* New York: G. P. Putnam's Sons.

*The Saint Louis Medical and Surgical Journal.* Saint Louis: Owens Printing Company.

*The Therapeutic Gazette.* Detroit: George S. Davis.

*Quarterly.*

*The Alienist and Neurologist.* St. Louis: Ev. E. Carreras.

*Yearly.*

*Annual of the Universal Medical Sciences.* Philadelphia: F. A. Davis.

*Transactions of the American Surgical Association.* Philadelphia: P. Blakiston, Son & Co.

*Occasionally.*

*Transactions of the New York Academy of Medicine.* New York: 12 West 31st Street.

### ASIA.

### CHINA.

*Quarterly.*

*The China Medical Missionary Journal.* Shanghai: Kelly & Walsh, Limited.

### INDIA.

*Monthly.*

*The Indian Medical Gazette.* Calcutta: Thacker, Spink & Co.

### JAPAN.

*Monthly.*

*The Sei-I-Kwai Medical Journal.* Tokio: Z. P. Maruya & Co.

### AUSTRALASIA.

### AUSTRALIA.

*Monthly.*

*The Australasian Medical Gazette.* Sydney: L. Bruck.

*The Australian Medical Journal.* Melbourne: Stillwell & Co.

### NEW ZEALAND.

*Quarterly.*

*The New Zealand Medical Journal.* Dunedin: J. Wilkie and Co.

### EUROPE

### BELGIUM.

*Monthly.*

*Bulletin de l'Académie royale de médecine de Belgique.* Brussels: F. Hayez.

### BRITISH ISLES.

*Weekly.*

*British Medical Journal.* London: 429 Strand.

*The Hospital.* London: 140 Strand.

*The Lancet.* London: 234 Strand.

*Monthly.*

*The Birmingham Medical Review.* Birmingham: Hall & English.

*The Journal of Laryngology and Rhinology.* London: F. A. Davis.

*The Medical Chronicle.* Manchester: John Heywood.

*The Practitioner.* London: Macmillan & Co.

*Edinburgh Medical Journal.* Edinburgh: Oliver and Boyd.

*The Glasgow Medical Journal.* Glasgow: Alex. Macdougall.

*The Dublin Journal of Medical Science.* Dublin: Fannin & Co.

*Quarterly.*

*Archives of Surgery.* London: J. & A. Churchill.

*The Asclepiad.* London: Longmans, Green, & Co.

*The British Gynæcological Journal.* London: John Bale & Sons.

*Half-yearly.*

*The Liverpool Medico-Chirurgical Journal.* Liverpool: Medical Institution.

*The Retrospect of Medicine.* London: Simpkin, Marshall, & Co.

*Yearly.*

*Guy's Hospital Reports.* London: J. & A. Churchill.

*St. Thomas's Hospital Reports.* London: J. & A. Churchill.

*The Directory of Second-hand Booksellers.* Rochdale: James Clegg.

*The Medical Annual.* Bristol: John Wright & Co.

*The Year-Book of Treatment.* London: Cassell & Company, Limited.

*Transactions of the Royal Academy of Medicine in Ireland.* Dublin: Fannin & Co.

*Year-Book of Scientific and Learned Societies.* London: C. Griffin & Co.

DENMARK.

*Weekly.*

*Hospitals-Tidende.* Copenhagen: Jacob Lund.

FRANCE.

*Three times a week.*

*Gazette des hôpitaux.* Paris: 4 Rue de l'Odéon.

*L'Union médicale.* Paris: 11 Rue de la Grange-Batelière.

*Weekly.*

*Bulletin de l'Académie de médecine.* Paris: G. Masson.

*Le Progrès médical.* Paris: 14 Rue des Carmes.

*Twice a month.*

*Gazette de Gynécologie.* Paris: O. Doin.

*Monthly.*

*Revue mensuelle de Laryngologie, d'Otologie et de Rhinologie.* Paris: 8 Place de l'Odéon.

GERMANY.

*Weekly.*

*Deutsche medicinische Wochenschirft.* Berlin: Georg Thieme.

*Medicinische Bibliographie.* Leipzig: Breitkopf & Härtel.

*Monthly.*

*Fortschritte der Krankenpflege.* Berlin: H. Kornfeld.

*Quarterly.*

*Bibliotheca Medico-Chirurgica.* Gottingen: Vandenhoeck & Ruprecht.

GREECE.

*Weekly.*

*Γαληνὸς.* Athens: N. G. Passare.

HOLLAND.

*Weekly.*

*Nederlandsch Tijdschrift voor Geneeskunde.* Amsterdam: F. van Rossen.

ITALY.

*Twice a month.*

*Lo Sperimentale.* Florence: 35 Via degli Alfani.

NORWAY.

*Monthly.*

*Norsk Magazin for Lægevidenskaben.* Christiania: Th. Steens.

PORTUGAL.

*Weekly.*

*A Medicina Contemporanea.* Lisbon: Jose Antonio Rodrigues & Ca.

ROUMANIA.

*Twice a month.*

*Spitalul.* Bucharest: Stefan Mihalescu.

RUSSIA.

*Weekly.*

*Medycyna.* Warsaw: 80 Allée de Jérusalem.

*Vrach.* St. Petersburg: C. Ricker.

SPAIN.

*Weekly.*

*El Siglo Médico.* Madrid: Magdalena, 36, 2º

*Twice a month.*

*Revista de Ciencias Médicas.* Barcelona: 9 Calle Fortuny.

SWEDEN.

*Quarterly.*

*Nordiskt Medicinskt Arkiv.* Stockholm: P. A. Norstedt & Söner.

TURKEY.

*Twice a month.*

*Gazette Médicale d'Orient.* Constantinople: Edgar Whitaker.

